# From water to land: Evolution of photoreceptor circuits for vision in air

**DOI:** 10.1371/journal.pbio.3002422

**Published:** 2024-01-22

**Authors:** Tom Baden

**Affiliations:** University of Sussex, Sussex Neuroscience, Sussex Center for Sensory Neuroscience and Computation, Brighton, United Kingdom

## Abstract

When vertebrates first conquered the land, they encountered a visual world that was radically distinct from that of their aquatic ancestors. Fish exploit the strong wavelength-dependent interactions of light with water by differentially feeding the signals from up to 5 spectral photoreceptor types into distinct behavioural programmes. However, above the water the same spectral rules do not apply, and this called for an update to visual circuit strategies. Early tetrapods soon evolved the double cone, a still poorly understood pair of new photoreceptors that brought the “ancestral terrestrial” complement from 5 to 7. Subsequent nonmammalian lineages differentially adapted this highly parallelised retinal input strategy for their diverse visual ecologies. By contrast, mammals shed most ancestral photoreceptors and converged on an input strategy that is exceptionally general. In eutherian mammals including in humans, parallelisation emerges gradually as the visual signal traverses the layers of the retina and into the brain.

## Abstract 2

When vertebrates first conquered the land, they encountered a visual world that was radically different from that of their aquatic ancestors. This Essay looks at how eyes evolved to cope with the move from seeing through water to seeing through air.

## Introduction

**Fig 1:**
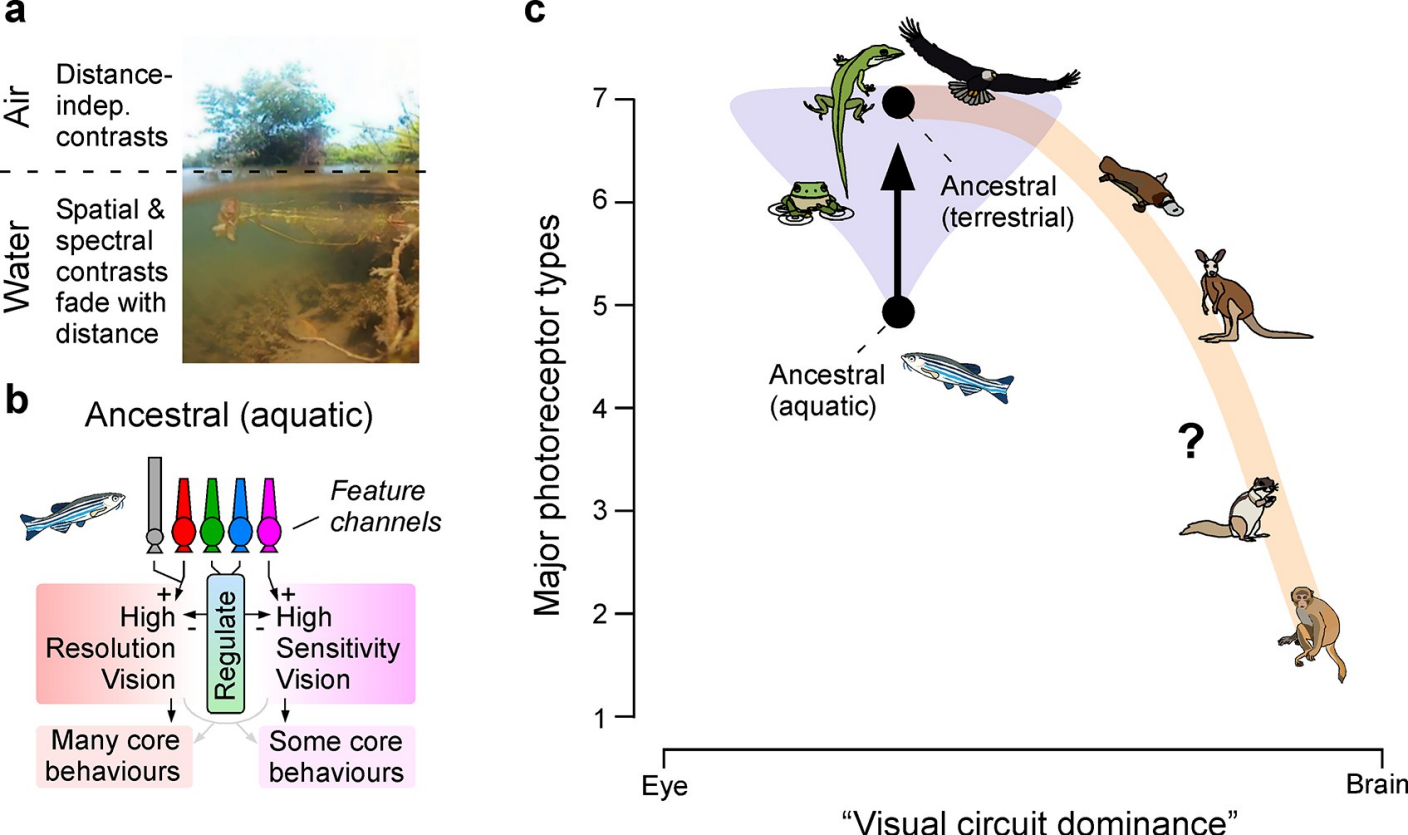
Conceptual summary of retinal circuit strategies across vertebrates. (**a**) Split-photo showing a riverine underwater scene and its corresponding view above the surface. Note that below the water, but not above, visual structure rapidly disappears with distance. (**b**) Retinal circuit summary of how ancestral photoreceptor types (cf. Fig 2A) probably serve as parallel feature channels that differentially drive and/or regulate into distinct sets of behavioural programmes (from ref [1]). (**c**) Conceptual summary of how early terrestrial vertebrates expanded the 5 ancestral aquatic retinal input channels to 7. Most extant tetrapods use variants of this “ancestral terrestrial” strategy, but mammals gradually shed photoreceptor diversity while offloading visual computations from the retina to the brain. Animal schematics taken from ref [122].

Vertebrate vision first evolved in the water, where for more than 150 million years it was consistently based on the signals from 5 anatomically and molecularly distinct types of photoreceptor neurons: rods, as well as ancestral red, green, blue, and UV cones (expressing RH, LWS, RH2, SWS2, and SWS1 opsin, respectively) [1–3]. In the water, these 5 input streams are probably best thought of as parallel feature channels that deliver distinct types of information to distinct downstream circuits [1]. This is because water absorbs and scatters light in a wavelength-dependent manner (Fig 1A), which means that “beyond colour” [1], different spectral photoreceptor channels inherently deliver different types of visual information.

As argued in a recent perspective [1], aquatic visual systems evolutionarily reached solutions that exploit these differences. In this view, photoreceptors represent parallel channels that are differentially wired to drive and/or regulate distinct behavioural programmes (Fig 1B): First, rods and ancestral red cones are the eyes’ primary brightness sensors. They are used for general purpose vision and to drive circuits for body stabilisation and navigation. Second, ancestral UV cones are used as a specialised foreground system, primarily wired into circuits related to predator–prey interactions and general threat detection. Third, ancestral green and blue cones probably represent an auxiliary system, tasked with regulating rather than driving the primary red/rod and UV circuits.

This ancestral strategy exploits the specific peculiarities of aquatic visual worlds; however, in air the same rules do not necessarily apply. For example, in the water, object vision can be a relatively easy task, because background structure tends to be heavily obscured by an approximately homogeneous aquatic backdrop [4]. At short wavelengths including in the UV range, this effect can be so extreme that no background is visible at all [5]. Many small fish exploit this fact of physics to find their food [5–9]. Above the water, this and many other “ancestral visual tricks” no longer work, because in air, contrast tends to be largely independent of viewing distance: Everything is visible at high contrast [10]. Accordingly, when early would-be tetrapods started to peek out of the water, strong selection pressures would have favoured a functional reorganisation of some of these inherited aquatic circuits, and nowhere is this more evident that at the level of the photoreceptors themselves.

One of the earliest and perhaps most important retinal circuit changes was the emergence of the double cone [1,11], which took the “aquatic ancestral” photoreceptor complement of 5 to a “terrestrial ancestral” complement of 7 (Fig 1C). The visual systems of all extant tetrapods, including humans, directly descend from this early “terrestrialised” retinal blueprint. However, from here, different descendant lineages have taken this highly parallelised retinal input strategy and embarked upon radically different visual paths. Most lineages, including those that led to modern-day amphibians, reptiles, and birds, have retained the terrestrialised ancestral blueprint, modifying upon it to suit their unique visual ecologies. Mammals, however, have ended up on a very different path. Their early synapsid ancestors gradually shifted some of their visual systems’ heavily lifting out of the eye and into the brain. Along this path—whether as cause or consequence—descendant lineages gradually reduced their photoreceptor complements from 7 types to 6, then 5, and eventually to the mere 3 that we see in modern-day eutherians (Fig 1C) [1]: Rods (RH), as well as ancestral red (LWS) and UV cones (SWS1).

Primates including humans have then then taken this eutherian strategy to the extreme: More than 99.9% of all photoreceptors in our eyes are either rods or ancestral red cones (including both “red-” and “green-shifted LWS variants”) [12], the ancestral “general purpose” system of the eye. The remaining 0.1% is what is left of the ancestral UV system, today expressing a blue-shifted variant of the SWS1 opsin [3] (hence often called “blue cones,” not to be confused with ancestral blue cones that express SWS2). In concert, the “three” cone variants drive achromatic vision (although with limited contribution from ancestral UV cones), while in opposition they serve colour vision [13]. However, this “textbook strategy” is far removed from the original aquatic circuit design and probably quite unique to our own lineage [1]. Accordingly, for understanding vision in a general sense, and to understand our own visual heritage, it will be critical to pay homage to vertebrate’s shared evolutionary past. Here, vision is built on a retinal circuit design that begins with major parallelisation right at the input.

### From water to air

**Fig 2:**
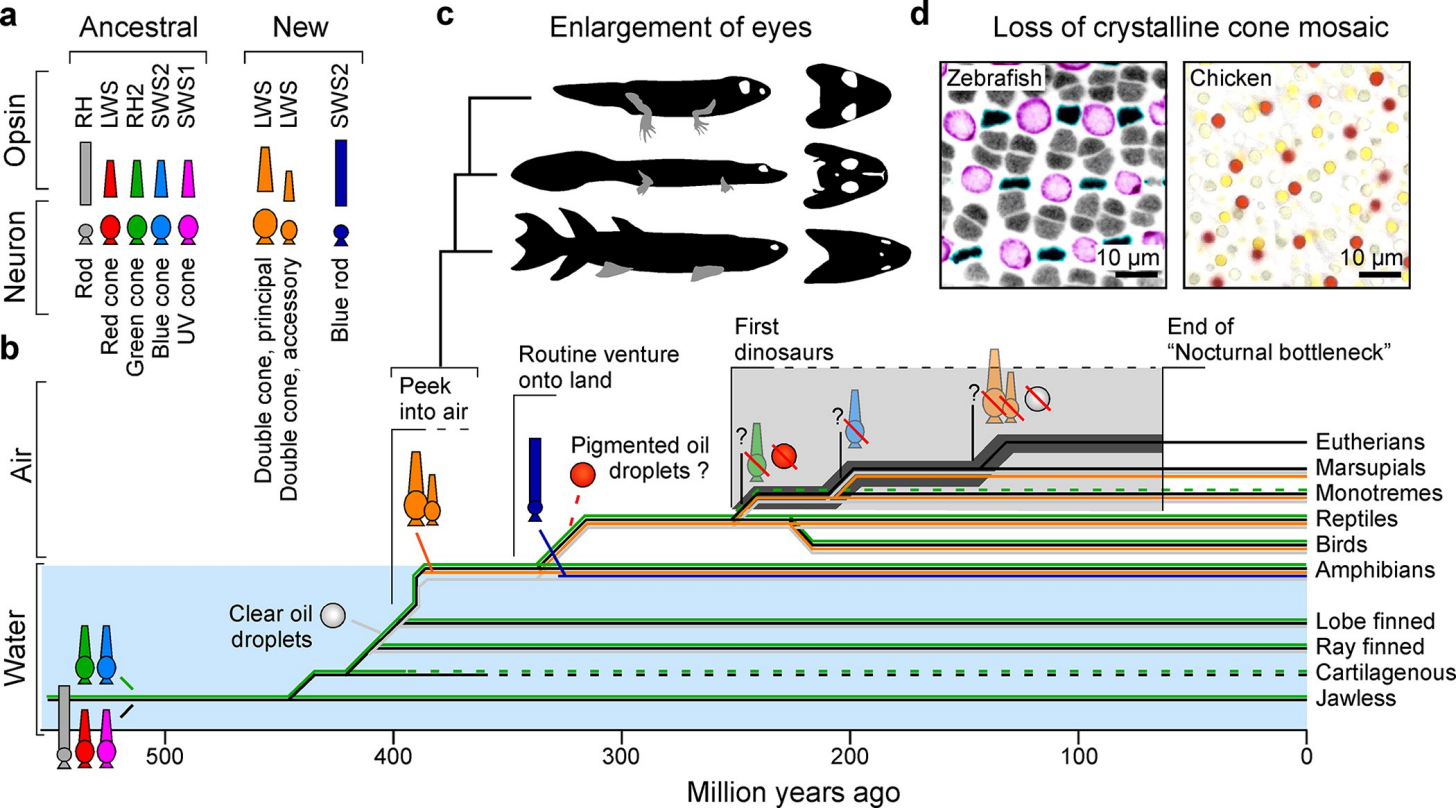
Vertebrate photoreceptors over evolutionary time. (**a**) Summary of vertebrate photoreceptor neurons (bottom) and their typically expressed opsins (top), including the 5 ancestral types (left) and the 3 “new” types that emerged soon after early tetrapods’ colonisation of the land. (**b–d**) Approximate timeline of vertebrate evolution and key changes in eyes and photoreceptor complements as indicated (cf. Fig 1D). Schematised reconstruction of early tetrapod skull shapes illustrate changing body shapes and eye enlargements (c, based on ref [14], from bottom: Eusthenopteron, Acanthostega, Pederpes). Zebrafish and chicken cone mosaics (d) modified from refs [123] and [124], respectively.

Following more than 150 million years of aquatic vision, vertebrates started to peek above water surface in the Devonian, some 390 mya [10,14]. This would change everything. In air, water’s strong scatter and absorption of light are essentially gone, and this would have (i) provided more photons for vision overall; (ii) disabled water’s links between spectral content and viewing distance; and (iii) made it possible to see for kilometres rather than metres. The expansion in visual interaction range may have driven the expansion of brain complexity in terrestrial species [15], for example, because this would have turned visual predator–prey interactions from largely fast and reactionary encounters to ones that necessitated long-term planning. However, not only brains became more complex: So did eyes [16] (Fig 2A–2D)!

The eyes of early tetrapods differed from the ancestral aquatic eyes of fish in at least 3 important ways: Eyes were substantially larger [14] (Fig 2C), the previously crystalline cone mosaic of fish [17,18] was replaced with a locally random mosaic [19,20] (Fig 2D), and new types of photoreceptors emerged [11,21,22] (Fig 2A and 2D). The order of these events or their possible interdependencies are difficult to reliably reconstruct today, but all 3 traits emerged before the subsequent split of amphibians, some 340 mya.

#### Eye enlargement

This transition, which preceded the emergence of well-developed limbs and digits [14], is chiselled into the fossil record: Within only a few million years, early lobe-finned fish transitioned from a swimming form with small eyes, to an “underwater walking” form with large eyes (Fig 2C). Our last fully aquatic ancestors probably lived in the shallows but peeked out of the water in search of food. Invertebrates had colonised the land some 50 million years prior [23], and with no vertebrate predators around, their initial lack of defences [24] would have made for an easy meal. While tetrapod eye enlargement was therefore likely an adaptation for a crocodilian-like lifestyle, it opened the floodgates for much to come.

#### Shattering the cone mosaic

Many surface dwelling fish have a crystalline cone mosaic with fixed photoreceptor-stoichiometry of 2:2:1:1 for red, green, blue, and UV, and with rods slotted around UV cones [4,17,18] (Fig 2D, left). In species where subsets of cone types are missing the mosaic rearranges accordingly to close any gaps [17,25]. The functional purpose of this crystalline mosaic remains poorly understood; however, it may confer important computational advantages, perhaps not too dissimilar to some of those conferred by the crystalline organisation of crustacean eyes [26], which notably includes insects [27–29]. Conceivably, the mosaic might also be exploited to reliably match “driving” and “regulatory” cone circuits (cf. Fig 1B). In support, in some fish the crystalline lattice propagates onto subsets of horizontal and bipolar cells [17,30].

However, the crystalline solution of fish does not work in air. First, it would lead to spatial oversampling in the periphery, because unlike in water, terrestrial optics cannot simultaneously maintain perfect focus across the entire back of the eye [31]. Second, due to greatly reduced photon scatter and absorption in air, everything is visible with high contrast for as far as the eye can see. Sampling such an environment with a regular cone lattice leads to aliasing [32], which would in turn incapacitate motion circuits. This problem is long recognised in digital photography where it is solved by introducing blur; however, this solution is energy inefficient. In biology, a better option is to break the crystalline arrangement in favour of a locally randomised pattern (Fig 2D, right). This also opened up the possibility to discard the fixed cone stoichiometry in favour of distinct and locally flexible cone ratios that best serve an animal’s specific visual ecology—as is the case in all extant tetrapods [3,20].

And yet, one mystery remains: Extant lobe-finned fishes, the closest living relatives to the aquatic ancestors of tetrapods, have no crystalline mosaic [17,33,34]. The arrangement is also not found in the “preceding” cartilaginous fish or in lampreys [17,35]. The crystalline pattern may therefore be a clade-specific trait of ray-finned fishes that is not ancestral to the tetrapod eye. Alternatively, the crystalline arrangement could have evolved in the common ancestor to all teleosts but have subsequently been lost in extant lobe-finned species, of which there are only 2 small lineages: coelacanth, who live in the deep where light is limiting [36] and where the mosaic breaks also in ray-finned fish [37], and lungfish [33,34], who like early tetrapods routinely peek out of the water.

#### New photoreceptors

At least 2 new sets of photoreceptor types emerged in early tetrapods: “double cones” [11] and “blue rods” [21,38,39]. Both are present in amphibians but absent in fish, suggesting that their emergence coincided with the first major presence of vertebrates on land. Correspondingly, their purpose is probably related to exploiting new opportunities that presented themselves above the water surface.

Of the 2 new photoreceptor systems, double cones probably appeared first. They exist in amphibians [39], reptiles [40,41], birds [11,22], monotremes [42–44], and marsupials [44–46], and therefore probably appeared in a common ancestor to all these lineages: the first tetrapods (Figs 2B and 1C, cf). They are again missing in eutherian mammals [3]. By contrast, blue rods are only present in amphibians [21,38] and therefore probably emerged after their lineage had already diverged from that of subsequent terrestrial species. The origin of double cones is debated, but their anatomical similarity to pairs of ancestral red and green cones in the eyes of fish [18] points at their joint duplication as a likely candidate. Pairs of teleost red and green single cones are also sometimes referred to as double cones [47]; however, this is a historical misnomer: Fish only have 1 set of these cones, while non-eutherian tetrapods have 2: the ancestral set of red and green single cones, plus an independent population of double cones.

#### Duplicating red/green circuits?

Double cones are made up of 2 tightly associated photoreceptors, called the principal and accessory member [11]. In line with its putative origin from the red single cone, the principal member expresses the red LWS opsin, but the same opsin is also usually [48] found in the accessory member [22]. The latter may have resulted from an opsin expression switch from RH2 to LWS. The 2 members of the double cone also differ in their oil droplets [49,50] (see below).

Beyond inheriting opsins and morphological characteristics, any newly duplicated cones would presumably also have inherited their ancestors’ postsynaptic circuitry. This would have opened the option to share or take over some of their original functions. In agreement, just like red and green single cones, the 2 members of the double cone feed into mutually independent horizontal and bipolar cell circuits [11]. Conceivably, the ancestral one-size-fits-all strategy of using red cones to drive most of vision could therefore now be broken into 2 independent circuits, each with their own regulatory system. This in turn would have enabled individual specialisation of the 2 circuits for different sets of visual tasks. One of these tasks, at long last, might have been the emergence of a dedicated system for colour vision as we think of it today.

#### Co-option of ancestral single cones for terrestrial colour vision?

This view is supported by observing phylogenetic patterns in photoreceptor oil droplets—small optical elements that are positioned immediately in front of cones’ light sensitive outer segments [49]. Oil droplets are common in vertebrates, from lobe-finned fish to marsupials, and again absent in eutherians. Moreover, some ray-finned fish have ellipsosomes, which though developmentally distinct, appear functionally reminiscent of oil droplets [51]. In general, oil droplets can serve many functions, but one of them is spectral sharpening [49,52,53]. By mixing coloured pigments into the oil, the droplets can be used as filters that restrict a cone’s spectral sensitivity. This limits spectral overlap between cones and thereby increases colour resolution. However, this sharpening comes at the cost of reduced light sensitivity, which means that it is not possible to simultaneously optimise the same photoreceptor for high signal to noise greyscale vision and for spectrally narrow colour vision. Prior to the emergence of double cones, this trade-off would have precluded ancestral red cones from using oil droplets (or ellipsosomes) in this way, and correspondingly, all teleosts [49] except lungfish [33,34] use them for non-spectral tasks. Conversely, oil droplets are often heavily pigmented in red cones of amniotes, while double cone droplets remain at most weakly pigmented, and sometimes absent [49,52,53]. Together, this hints that double cones, led by the principal member and regulated by the accessory member, took over some of the original roles of the long wavelength system in supporting high acuity spatiotemporal tasks [54]. This could have freed up the use of single cones for spectral vision.

**Fig 3:**
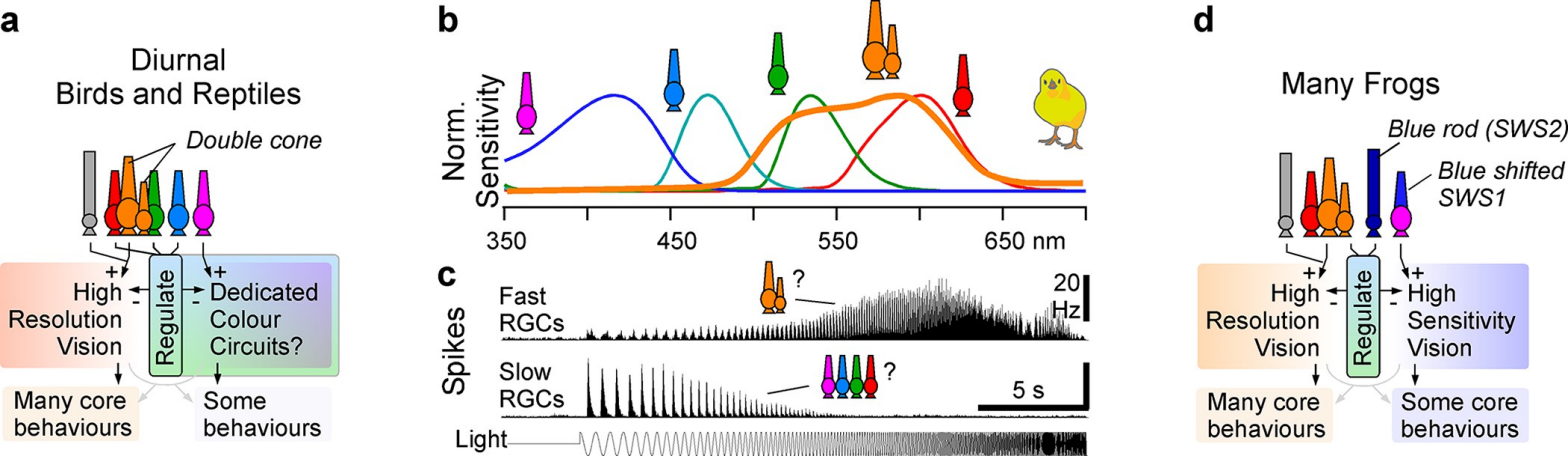
Retinal circuits of nonmammalian tetrapods. (**a**) Conceptual retinal wiring schema of diurnal birds and reptiles, illustrating how double cones might fit into the circuit blueprint inherited from fish (cf. Fig 1B). While double cones might partially take over greyscale functions of the ancestral red system, existing and new interactions between single cone circuits might have increasingly been coopted for colour vision. (**b**) Spectral sensitivity functions of chicken cones with oil droplets included (based on ref [125]). (**c**) Mean spike rates of 2 functionally identified ganglion cell clusters from poultry chick retina in response to a time-accelerating full field chirp stimulus as indicated (mod. from ref [65]). (**d**) As (a), but for frogs, who lost ancestral green cones (RH2) but evolved “blue rods.” Potentially, blue rods serve functions similar to those served by ancestral blue cones of fish, however at lower light levels.

In agreement, most diurnal birds retain the full complement of ancestral single cones, and these cones’ interplay with oil droplets leads to the probably most highly resolved colour vision system among vertebrates [22,49,52,53] (Fig 3A and 3B). Double cones are theoretically not needed for this task [54]. Among single cones, any surviving opponency of ancestral green and blue circuits [55] would no longer be overly useful for estimating distance in air [1,10,56], and similarly, their requirements for regulating motion circuits likely changed [57]. This would have opened the possibility to co-opt some of their existing, spectrally nuanced retinal and central circuits for new functions, such as colour vision. Likewise, some of the ancestral functions of UV cones, such as prey capture, are less applicable in air, and probably opened further options for spectral specialisations. A decreased dependence on single cones for supporting achromatic vision, alongside the no longer fixed stoichiometry as necessitated in fish, would have allowed retinal circuits to reduce their relative abundances, including of their postsynaptic circuits. For example, in chicken only around 20% of cones are ancestral blue- or UV cones [19], and these feed into fewer than 10% of bipolar cells [11], down from more than half in zebrafish [58]. This reduction in short wavelength contributions to retinal circuits is pervasive across terrestrial species and taken to the extreme in our own eye: Fewer than 0.1% of photoreceptors in the human retina are ancestral UV cones, and only a single type of bipolar cell specifically represents their signals [59,60].

#### Double cones took over (some of) achromatic vision

While single cones potentially specialised for colour vision, double cones may have specialised for high acuity spatiotemporal vision [54,61]. In chicken, they co-wire with rods [11], the ancestral remit of red single cones [58,62]. Double cones are usually also the largest and most numerous cone types, which maximises their potential for temporal and spatial resolution, respectively [63]. In support, birds generally have some of the fastest eyes of any vertebrate [64], and the spectral tuning of birds’ fastest retinal output channels (Fig 3C) is consistent with a primary drive from a red opsin expressing cone [65]. However, the extent to which these fast channels are driven by red single and/or double cones remains unknown. It is also unclear if the principal and accessory members of the double cone work jointly (as suggested by their intimate coupling), in mutual opposition (as hinted by their putative ancestry), or both. Despite their pervasive presence across vertebrates, from frogs [39] to kangaroos [44], systematic data on the function of double cones and their downstream circuits remains close to nonexistent.

Moreover, despite the newfound potential for differentially specialising red circuits, some important aspects of ancestral red single cones for driving achromatic vision must have been retained: First, diurnal raptors have the highest spatial resolution of any vertebrate, but in most species, this is achieved without the use of double cones which are systematically absent from their foveae [66]. Second, eutherian mammals lost the double cone, but they can see perfectly fine. If double cones would have completely taken over general-purpose greyscale vision in the ancestors of eutherian mammals, our own eyes today would probably be covered in double cones, not red single cones.

#### Extending red regulation into the night

While regulation of red and UV functions by mid-wavelength circuits may work well during the day (Fig 1B), the strategy has 1 major flaw: Any regulation would cease as soon as rods take over, because most species have no “second rod” to regulate the first one. In fish, this absence for the potential to regulate rods may in part reflect a compromise driven by the reduced light levels in the water compared to in air. As photons become sparse, visual systems increasingly struggle with signal to noise more than they do with the issues that putatively drove the evolution of regulatory systems in the first place [67,68]. However, with the appearance of early amphibians, this pressure may have lifted: Amphibians must live near or above the water surface where there is more light, including at night. They also have proportionally larger eyes compared to most surface-dwelling fish that share parts of their habitat [69], and their rods are some of the largest among vertebrates [21]. All these factors contribute to improving night vision, and this in turn might have opened a path towards rod-regulation (Fig 3D), much like vertebrates’ earliest fully aquatic ancestors might have done some 300 million years prior for the cone system. Moreover, unlike most other tetrapods, amphibians routinely return to the water where the previously discussed spectral features [1] still apply.

The origin of amphibian “blue-rods” remains debated [21,70]. Its rod-like features (e.g., [71]) hint that it might reflect a duplication of the ancestral “green” rod, while its expression of the SWS2 opsin instead points to its duplication from blue cones. In general, cones can adopt rod like features over time—even the ancestral “green” rod (RH1) may represent an ancient duplication of green cones (RH2) [72]. Similarly, some snake [73] and gecko [74] photoreceptors are thought to have switched back and forth between rod- and cone-like features as their lineages iteratively adopted diurnal and nocturnal lifestyles. Paralleling the possible duplication of the red-green system to yield double cones, a blue cone origin of “blue rods” would also be in line with their possible regulation of green rod circuits (Fig 3D). However, as with double cones, the purpose and functional substrate of “blue-rod” circuits remain sparsely explored. Nevertheless, anatomical work hints that blue rods do make outer retinal connection that differ from those of ancestral rods [21,39], and the 2 rod systems can be differentially read out at the level of behaviour [70].

Together, the 2 new major photoreceptor systems that emerged well-timed with the first long-term emergence of vertebrate life on land might represent a near-complete duplication of the full ancestral cone complement. That is, except for UV. Plausibly, there was little selection pressure for duplicating this system because the background subtraction effect offered by aquatic UV-vision no longer applies in air. Accordingly, UV cones would have become less useful for supporting basic figure ground segmentation tasks including prey capture. Their utility in threat detection, however, would remain [75,76].

### What happened in mammals?

#### Revisiting the nocturnal bottleneck

From early jawless species via fish and amphibians to present-day reptiles and birds, vertebrate retinas have increased considerably in their capabilities over their more than 500-million-year histories (Fig 2B). And yet, much of this ancestral accumulation of complexity is lost in our own lineage. Unlike even our still relatively close marsupial “cousins,” retinas of eutherian mammals have shed most of the early inventions. Gone are 2 of the original 4 ancestral single cones, alongside both double cones, and oil droplets are not to be found anywhere from mice to men. Even ancestral UV cones, though present, appear to play an at best peripheral role in shaping the visual world of eutherians [16].

The series of loss events detailed in the above have long been linked to Walls’ “nocturnal bottleneck theory” [77]. With the appearance of diurnal predatory dinosaurs some 240 million years ago, early ancestors of today’s eutherian mammals were forced into a nocturnal niche. At night, less light is available for vision, and this would then have gradually led to the loss of all 4 photoreceptor systems (both double cones as well as green and blue single cones), and oil droplets along with it. However, it might be time to revisit this idea. The only major mammalian lineage to have systematically lost these photoreceptors are eutherians, which diverged from marsupials only some 170 million years ago [78]. However, marsupials retain both double cones and oil droplets [45,46,79], indicating that these features must have been retained in the last eutherian ancestor for at least 70 million years of previous nocturnalisation. Similarly, monotremes, diverging some 220 mya, retain the ancestral blue cone [42,43]. Accordingly, blue cones were retained for at least 20 million years of nocturnalisation. Green cones present a further conundrum. The absence of the green cone’s RH2 gene in the genome of all mammals, including monotremes and marsupials, suggests that they were lost early on [80]. And yet, some marsupials including dunnarts have a “third” unaccounted for mid-wavelength sensitive cone [45,46,81]. The identity and origin of this “extra” cone remains debated among 2 perhaps equally plausible options: Either, this is the ancestral green cone, but now expressing a non-RH2 opsin, or it is the result of yet another cone-duplication. In either case, already the retention of blue cones for at least 20 million years of sustained nocturnalisation, and of double cones plus oil droplets for an additional 50, poses a serious challenge for the nocturnal bottleneck as the main driving force for cone loss in the ancestors of eutherian mammals. Instead, it seems reasonable to suggest that other factors contributed, at least in part. One of these factors might be related to their “other half” of vision: the brain itself (cf. Fig 1C)!

#### Eutherian mammals may use a different retinal “code”

Mammals have some of the largest brains relative to body size of all vertebrates, with eutherians somewhat outcompeting marsupials and monotremes [82,83]. The concomitant expansion in brain complexity might well have allowed emerging central circuits to take over some of the more complex computations that were previously supported by the retina. Over evolutionary time, and particularly upon their emergence on land, vertebrates’ increasingly complex visual requirements perhaps inevitably outran the increasing capacity of retinal circuits to deliver an immediately actionable signal for brain circuits to use. Compared to our earliest sighted ancestors, some degree of centralisation of key visual tasks has probably occurred in all extant vertebrates. Mammals, however, seem to have embarked upon this path more than any other lineage. In eutherians, vision has pivoted to a point where it is driven almost exclusively by ancestral red cones and rods, the original “general purpose system” of the vertebrate eye. From a retinal perspective, this strategy is relatively simple, general purpose, and probably efficient. After all, vertebrate retinal complexity—with a pinnacle probably in birds—is perhaps not the only option for building a powerful and versatile visual system.

Based on our still very limited understanding of retinal coding outside of mammals, the “visual way” of eutherians appears to be diametrically opposite to that of most fish and their diverse non-eutherian descendants including birds [65]. Eutherians tend to represent On and Off or fast and slow signals via parallel streams, with only a minority of neurons occupying the coding space in between these extremes [65,84–87]. Conversely, in chicken at least [61], the output from the avian retina is dominated by a highly correlated “compound code” that routinely mixes On and Off as well as fast and slow signals within individual ganglion cell axons [65]. Overall, the eutherian retina strategy therefore appears to be one of decorrelation [88,89], likely to achieve coding efficiency by keeping axonal firing rates low [90–94]. In birds, firing rates are probably higher on average, which hints their retinal code is not very energy efficient [65]. It is, however, space efficient, in the sense that multiple messages can be “multiplexed” via common axons. Conceivably, this avian strategy is a consequence of their anatomically much more dense retinal input-output organisation and enabled by a 3-fold lower energy consumption per neuron compared to those of mammals [95].

**Fig 4:**
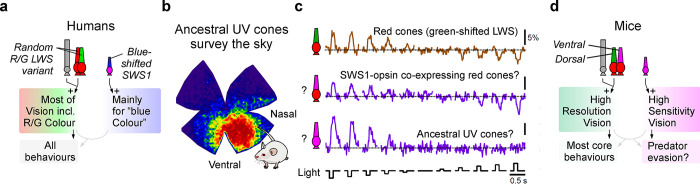
Retinal circuits of eutherian mammals. (**a**) Conceptual circuit model for human vision, based on modifications to the ancestral blueprint of fish (cf. Fig 1B). In humans, most cones are ancestrally red, but they randomly express a “red” or a “green-shifted” variant of the LWS opsin (ancestral red cones expressing the “green-shifted” LWS variant are often called green cones. They are not to be confused with ancestral green cones which express RH2, and which are lost in all mammals). A small number of ancestral UV-cones are also retained, now expressing a “blue-shifted” variant of SWS1, hence called blue cone (not to be confused with ancestral blue cones). (**b**) Retinal distribution of ancestral UV cones in mouse (mod from ref [76]). (**c**) Example linear (top/middle) and dark-biased (bottom) contrast responses of mouse cones (mod from ref [75]). Putatively, the dark biased responses can be attributed to ancestral UV cones, while the linear responses could come from ancestral red cones. (**d**) As (a), but for mice.

As for eutherian mammals, nowhere is their “spring-cleaned” retinal output strategy more obvious than in the case of humans and our closest nonhuman primate relatives. More than 80% of the retinal fibres leaving our eyes, leading to a probably even greater fraction of our conscious visual experience, come from just 2 pairs of projection systems: midgets and parasols [60,96,97]. Midgets are numerous and tiny and provide exquisite spatial resolution, while parasols are larger which allows them to pool cone signals, ultimately serving fast temporal resolution. The “code,” delivered to the cortex via the thalamus, is exceptionally general and flexible [91,98–101]. Even colour-signals are delivered via this route, and not using ancestrally distinct sets of cones. In humans, green and red signals are provided by the same ancestral red cone, with the tweak that some red cones randomly express a green-shifted version of the red opsin, while others express the “original” red version [59,102] (Fig 4A, left). During infancy, we learn the statistics of the consequently red- and green-biased signals as they come into the brain [103,104], and the resultant spectral performance in red-green discrimination appears to be second to none [98,105,106]. A fifth “major” type of retinal projection neuron, the small bistratified ganglion cell [59,107,108], additionally delivers spectrally opponent signals derived from contrasting ancestral red and UV cones, thereby essentially completing our trichromatic experience (Fig 4A, right). Beyond those 5 [97], other retinal output channels do exist, including some that resemble versions of ancestral circuits that once were probably fundamental to vertebrate’s success on this planet [59,109,110]. However, in the human eye, their abundance and diversity is low even when compared with mice [84,85,109], and their roles—for the most part at least—remain uncertain.

#### The remains of the ancestral UV system

Despite our overwhelming reliance on red cones and rods, some noncolour coding ancestral UV circuits may have survived. For example, visual snow syndrome can be exacerbated by selective UV cone activation [111]. This rare human condition has no obvious relation to colour vision—instead, it describes the tendency to experience perceptual noise—that is, to see a “snowy” image. The underlying causes remain poorly understood, but its potentiation following overexcitation of the ancestral UV system hints that it might be linked to primordial UV circuits that originally served other purposes [112].

Beyond the perhaps extreme case of humans, the much-studied retinas of rodents give further insights (Fig 4B–4D). Mice, for example, retain a patch of ancestral UV cones in their ventro-nasal retina, looking straight up at the sky [76] (Fig 4B). The function of this patch remains unclear, but one possibility is in aerial predator detection. Mice are in fact remarkable when it comes to UV vision [75,113–116] (Fig 4C and 4D). Dorsally, their photoreceptor complement resembles the “eutherian-standard” of “green-shifted” ancestral red cones plus rods, sparsely interspersed with UV cones. Ventrally, however, ancestral red cones are UV-sensitive because they co-express the UV-opsin [115]. Almost poetically opposite to the 2 types of red cones of birds, mice therefore have 2 types of UV-sensitive cones: the ancestral UV patch [76], embedded within a larger patch of UV co-expressing red cones [75,116]. This intermixed dual population of UV-sensitive cones has long left it unclear what ancestral UV cones contribute to this retina. After all, it makes little sense to simply “see UV twice”—unless, that is, if the 2 UV systems were used in distinct ways. The circuitry for doing so has presumably been in place for hundreds of millions of years. In support, even though all ventral mouse cones are UV sensitive, functionally they appear to fall into 2 distinct subpopulations [75]: One low gain and approximately linear—ideal for supporting general purpose vision—and another high gain with a strong dark bias—ideal for spotting the silhouettes of overhead birds against the sky [113,117] (Fig 4C). However, it remains unclear whether and how these 2 functional signatures are in fact linked to co-expressing versus ancestral UV cones, respectively (Fig 4D).

Beyond mice, co-expression of short wavelength opsins in ancestral red cones that “look” at the sky is remarkably common among terrestrial tetrapods [16,75,118]. If ancestral red cones are indeed the primordial general-purpose channel (Fig 1B), this awkward arrangement begins to make sense: Unlike in water, on land there is plenty of downwelling short wavelength light available, including at night [113,119,120]. In many cases, a UV image can in fact be substantially sharper compared to an equivalent red image [113,117,121], especially for detecting the edges of overhead vegetation [113,119]. Rather than using the ancestral UV cone complement to exploit this new opportunity, it was probably easier and therefore evolutionarily favourable to locally update the opsin driving the ancestral red circuit.

## Conclusions

Starting from their common aquatic ancestor [1], I have suggested a conceptual framework for tying together visual circuit strategies of extant tetrapods. Where eutherian mammals including humans represent one extreme of possible circuit organisations, birds probably represent the other. Mammals build their visual world from a relatively homogeneous population of input channels, gradually building complexity of feature representation as the signal ascends via retinal circuits and into the brain. Conversely, birds begin with a highly parallelised, and likely already complex feature representation at the level of the input, with for the most part unknown computational consequences for downstream circuits. Both strategies can work exceptionally well, as readily appreciated by simply observing visual behaviours of humans and eagles. However, how the 2 strategies can be linked at a circuit level that goes beyond the photoreceptors, and how other tetrapod lineages might fit into the greater picture, remains a major open question. Nevertheless, testable predictions abound, including some general areas of future investigation that may be particularly instructive:

- Can any of the “new” cone types be molecularly related to any of the old types (Fig 2), thereby potentially resolving their ancestry. How does these cones’ wiring compare to that of ancestral photoreceptors across species?
- What is the function of double cones and blue rods, and how are they structurally and functionally wired into the retina? Do all non-eutherian tetrapods use the same basic wiring strategy?
- What is the function and role of ancestral green and blue cones in terrestrial tetrapods that retain them, and how are they functionally routed onto behavioural programmes via the layers of the inner retina?
- What is the ancestral identity of the “third” cone of “trichromatic marsupials” [45], what is its function, and how is this cone wired into the retina? How structurally and functionally similar are the retinas of marsupials, who retain the double cone, to those of eutherian mammals, who lack it?

Addressing these, and many other loose ends promises steady progress on a path that may, in time, lead us to a truly general understanding of what it means for a vertebrate to “see,” how this is achieved at a circuit level, and in turn, how neural circuits and the computations they support evolve.

## References

[1] Baden T. Beyond Colour Vision: Ancestral photoreceptor diversity as the basis of visual behaviour. tbd. under review. 10.1038/s41559-023-02291-7 38253752

[2] Lamb TD. Evolution of phototransduction, vertebrate photoreceptors and retina. Prog Retin Eye Res. 2013:52–119. doi: 10.1016/j.preteyeres.2013.06.001 23792002

[3] Baden T, Osorio D. The Retinal Basis of Vertebrate Color Vision. Annu Rev Vis Sci. 2019;5:177–200. doi: 10.1146/annurev-vision-091718-014926 31226010

[4] Baden T. Circuit-mechanisms for colour vision in zebrafish. Curr Biol. 2021;31:PR807–R80. doi: 10.1016/j.cub.2021.04.053 34157269

[5] Yoshimatsu T, Schröder C, Nevala NE, Berens P, Baden T. Fovea-like Photoreceptor Specializations Underlie Single UV Cone Driven Prey-Capture Behavior in Zebrafish. Neuron. 2020;107:320–337.e6. doi: 10.1016/j.neuron.2020.04.021 32473094 PMC7383236

[6] Khan B, Jaesiri O, Lazarte IP, Li Y, Tian G, Zhao P, et al. Zebrafish larvae use stimulus intensity and contrast to estimate distance to prey. Curr Biol. 2023;0. doi: 10.1016/j.cub.2023.06.046 37437573

[7] Browman HI, Novales-Flamarique I, Hawryshyn CW. Ultraviolet photoreception contributes to prey search behaviour in two species of zooplanktivorous fishes. J Exp Biol. 1994;186:187–198. doi: 10.1242/jeb.186.1.187 9317606

[8] Novales FI. Opsin switch reveals function of the ultraviolet cone in fish foraging. Proc R Soc B Biol Sci. 2012;280:1–8. doi: 10.1098/rspb.2012.2490 23222448 PMC3574309

[9] Novales FI. Diminished foraging performance of a mutant zebrafish with reduced population of ultraviolet cones. Proc Biol Sci. 2016;283:20160058. doi: 10.1098/rspb.2016.0058 26936243 PMC4810871

[10] Nilsson D-E. Evolution: An Irresistibly Clear View of Land. Curr Biol. 2017;27:R715–R717. doi: 10.1016/j.cub.2017.05.082 28743021

[11] Günther A, Dedek K, Haverkamp S, Irsen S, Briggman KL, Mouritsen H. Double cones and the diverse connectivity of photoreceptors and bipolar cells in an avian retina. J Neurosci. 2021;41:5015–5028. doi: 10.1523/JNEUROSCI.2495-20.2021 33893221 PMC8197639

[12] Jacobs GH. Primate photopigments and primate color vision. Proc Natl Acad Sci U S A. 1996. doi: 10.1073/pnas.93.2.577 8570598 PMC40094

[13] Bowmaker JK. Evolution of colour vision in vertebrates. Eye Basingstoke. 1998. doi: 10.1038/eye.1998.143 9775215

[14] MacIver MA, Schmitz L, Mugan U, Murphey TD, Mobley CD. Massive increase in visual range preceded the origin of terrestrial vertebrates. Proc Natl Acad Sci U S A. 2017;114:E2375–E2384. doi: 10.1073/pnas.1615563114 28270619 PMC5373340

[15] Mugan U, MacIver MA. Spatial planning with long visual range benefits escape from visual predators in complex naturalistic environments. Nat Commun. 2020;11:3057. doi: 10.1038/s41467-020-16102-1 32546681 PMC7298009

[16] Baden T, Euler T, Berens P. Understanding the retinal basis of vision across species. Nat Rev Neurosci. 2020;21:5–20. doi: 10.1038/s41583-019-0242-1 31780820

[17] M-Ather A, Michel A. Retinas of Fishes: an Atlas. Berlin Heidelberg:Springer; 1976.

[18] Allison WT, Barthel LK, Skebo KM, Takechi M, Kawamura S, Raymond PA. Ontogeny of cone photoreceptor mosaics in zebrafish. J Comp Neurol. 2010;518:4182–4195. doi: 10.1002/cne.22447 20878782 PMC3376642

[19] Kram YA, Mantey S, Corbo JC, Hart N, Bowmaker J, Knowles A, et al. Avian cone photoreceptors tile the retina as five independent, self-organizing mosaics. Warrant E, editor. PLoS ONE. 2010;5:e8992. doi: 10.1371/journal.pone.0008992 20126550 PMC2813877

[20] Brainard DH. Color and the Cone Mosaic. Annu Rev Vis Sci. 2015;1:519–546. doi: 10.1146/annurev-vision-082114-035341 28532367

[21] Donner K, Yovanovich C. A frog’s eye view: Foundational revelations and future promises. Semin Cell Dev Biol. 2020. doi: 10.1016/j.semcdb.2020.05.011 32466970

[22] Kelber A. Bird colour vision–from cones to perception. Curr Opin Behav Sci. 2019;30:34–40. doi: 10.1016/j.cobeha.2019.05.003

[23] Shear WA, Edgecombe GD. The geological record and phylogeny of the Myriapoda. Arthropod Struct Dev. 2010;39:174–190. doi: 10.1016/j.asd.2009.11.002 19944188

[24] Wilson HM. Juliformian Millipedes from the Lower Devonian of Euramerica: Implications for the Timing of Millipede Cladogenesis in the Paleozoic. J Paleo. 2006;80:638–649.

[25] Carleton KL, Escobar-Camacho D, Stieb SM, Cortesi F, Justin MN. Seeing the rainbow: Mechanisms underlying spectral sensitivity in teleost fishes. J Exp Biol. 2020;223:jeb193334. doi: 10.1242/jeb.193334 32327561 PMC7188444

[26] Meyer-Rochow VB. The Crustacean Eye: Dark/ Light Adaptation, Polarization Sensitivity, Flicker Fusion Frequency, and Photoreceptor Damage. Zoolog Sci. 2001;18:1175–1197. doi: 10.2108/zsj.18.1175 11911074

[27] Groschner LN, Malis JG, Zuidinga B, Borst A. A biophysical account of multiplication by a single neuron. Nature. 2022;603:119–123. doi: 10.1038/s41586-022-04428-3 35197635 PMC8891015

[28] Wernet MF, Perry MW, Desplan C. The evolutionary diversity of insect retinal mosaics: Common design principles and emerging molecular logic. Trends Genet TIG. 2015;31:316–328. doi: 10.1016/j.tig.2015.04.006 26025917 PMC4458154

[29] Borst A, Groschner LN. How Flies See Motion. Annu Rev Neurosci. 2023;46:17–37. doi: 10.1146/annurev-neuro-080422-111929 37428604

[30] Mack AF. Evidence for a columnar organization of cones, Müller cells, and neurons in the retina of a cichlid fish. Neuroscience. 2007;144:1004–1014. doi: 10.1016/j.neuroscience.2006.10.029 17156929

[31] Navarro R. The Optical Design of the Human Eye: a Critical Review. Aust J Optom. 2009;2:3–18. doi: 10.3921/joptom.2009.3

[32] Kröger R. Anti-aliasing in image recording and display hardware: lessons from nature. J Opt Pure Appl Opt. 2004;6:743. doi: 10.1088/1464-4258/6/8/001

[33] Appudurai AM, Hart NS, Zurr I, Collin SP. Morphology, Characterization and Distribution of Retinal Photoreceptors in the South American (Lepidosiren paradoxa) and Spotted African (Protopterus dolloi) Lungfishes. Front Ecol Evol. 2016. doi: 10.3389/fevo.2016.00078

[34] Hart NS, Bailes HJ, Vorobyev M, Marshall NJ, Collin SP. Visual ecology of the Australian lungfish (Neoceratodus forsteri). BMC Ecol. 2008;8:1–14. doi: 10.1186/1472-6785-8-21 19091135 PMC2639370

[35] Magaña-Hernández L, Wagh AS, Fathi JG, Robles JE, Rubio B, Yusuf Y, et al. The functionally plastic rod photoreceptors in the simplex retina of Little skate (Leucoraja erinacea) exhibit a hybrid rod-cone morphology and enhanced synaptic connectivity. bioRxiv. 2023. p. 2023.06.28.546621. doi: 10.1101/2023.06.28.546621 PMC10614115 37827837

[36] Bruton MN, Stobbs RE. The ecology and conservation of the coelacanth Latimeria chalumnae. Environ Biol Fishes. 1991;32:313–339. doi: 10.1007/BF00007464

[37] de Busserolles F, Fogg L, Cortesi F, Marshall J. The exceptional diversity of visual adaptations in deep-sea teleost fishes. Semin Cell Dev Biol. 2020. doi: 10.1016/j.semcdb.2020.05.027 32536437

[38] Rozenblit F, Gollisch T. What the salamander eye has been telling the vision scientist’s brain. Semin Cell Dev Biol. 2020;106:61–71. doi: 10.1016/j.semcdb.2020.04.010 32359891 PMC7493835

[39] Nilsson S. An electron microscopic classification of the retinal receptors of the leopard frog (Rana pipiens). J Ultrastruct Res. 1964;10:390–416. doi: 10.1016/s0022-5320(64)80018-6 14188860

[40] Bowmaker JK, Loew ER, Ott M. The cone photoreceptors and visual pigments of chameleons. J Comp Physiol A Neuroethol Sens Neural Behav Physiol. 2005. doi: 10.1007/s00359-005-0014-4 16025336

[41] Loew ER, Fleishman LJ, Foster RG, Provencio I. Visual pigments and oil droplets in diurnal lizards: A comparative study of Caribbean anoles. J Exp Biol. 2002. doi: 10.1242/jeb.205.7.927 11916989

[42] Zeiss CJ, Schwab IR, Murphy CJ, Dubielzig RW. Comparative retinal morphology of the platypus. J Morphol. 2011;272:949–957. doi: 10.1002/jmor.10959 21567446

[43] Young HM, Pettigrew JD. Cone photoreceptors lacking oil droplets in the retina of the echidna, Tachyglossus aculeatus (Monotremata). Vis Neurosci. 1991;6:409–420. doi: 10.1017/s0952523800001279 2069895

[44] O’Day K. A Preliminary Note on the Presence of Double Cones and Oil Droplets in the Retina of Marsupials. J Anat. 1936;70:465–467. 17104608 PMC1249140

[45] Ebeling W, Natoli RC, Hemmi JM. Diversity of Color Vision: Not All Australian Marsupials Are Trichromatic. PLoS ONE. 2010;5:e14231. doi: 10.1371/journal.pone.0014231 21151905 PMC2997786

[46] Arrese CA, Hart NS, Thomas N, Beazley LD, Shand J. Trichromacy in Australian Marsupials. Curr Biol. 2002;12:657–660. doi: 10.1016/s0960-9822(02)00772-8 11967153

[47] Pignatelli V, Champ C, Marshall J, Vorobyev M. Double cones are used for colour discrimination in the reef fish, rhinecanthus aculeatus. Biol Lett. 2010. doi: 10.1098/rsbl.2009.1010 20129950 PMC2936199

[48] Beason RC, Loew ER. Visual pigment and oil droplet characteristics of the bobolink (Dolichonyx oryzivorus), a new world migratory bird. Vision Res. 2008;48:1–8. doi: 10.1016/j.visres.2007.10.006 18054982

[49] Toomey MB, Corbo JC. Evolution, development and function of vertebrate cone oil droplets. Front Neural Circuits. 2017;11:97. doi: 10.3389/fncir.2017.00097 29276475 PMC5727011

[50] Toomey MB, Collins AM, Frederiksen R, Cornwall MC, Timlin JA, Corbo JC. A complex carotenoid palette tunes avian colour vision. J R Soc Interface. 2015. doi: 10.1098/rsif.2015.0563 26446559 PMC4614492

[51] Nag TC, Bhattacharjee J. Retinal ellipsosomes: morphology, development, identification, and comparison with oil droplets. Cell Tissue Res. 1995;279:633–637. doi: 10.1007/BF00318176 7736559

[52] Vorobyev M, Osorio D, Bennett ATD, Marshall NJ, Cuthill IC. Tetrachromacy, oil droplets and bird plumage colours. J Comp Physiol—Sens Neural Behav Physiol. 1998. doi: 10.1007/s003590050286 9839454

[53] Bowmaker JK, Heath LA, Wilkie SE, Hunt DM. Visual pigments and oil droplets from six classes of photoreceptor in the retinas of birds. Vision Res. 1997. doi: 10.1016/s0042-6989(97)00026-6 9578901

[54] Lind O, Kelber A. The spatial tuning of achromatic and chromatic vision in budgerigars. J Vis. 2011;11:2–2. doi: 10.1167/11.7.2 21636524

[55] Yoshimatsu T, Bartel P, Schröder C, Janiak FK, St-Pierre F, Berens P, et al. Ancestral circuits for vertebrate color vision emerge at the first retinal synapse. Sci Adv. 2021;7:6815–6828. doi: 10.1126/sciadv.abj6815 34644120 PMC8514090

[56] Bartel P, Janiak FK, Osorio D, Baden T. Colourfulness as a possible measure of object proximity in the larval zebrafish brain. Curr Biol. 2021;31:R235–R236. doi: 10.1016/j.cub.2021.01.030 33689717 PMC7955152

[57] Mv Campenhausen, Kirschfeld K. Spectral sensitivity of the accessory optic system of the pigeon. J Comp Physiol A. 1998;183:1–6. doi: 10.1007/s003590050229

[58] Li YN, Tsujimura T, Kawamura S, Dowling JE. Bipolar cell-photoreceptor connectivity in the zebrafish (Danio rerio) retina. J Comp Neurol. 2012;520:3786–3802. doi: 10.1002/cne.23168 22907678 PMC3669674

[59] Dacey DM, Packer OS. Colour coding in the primate retina: Diverse cell types and cone-specific circuitry. Curr Opin Neurobiol. 2003;13:421–427. doi: 10.1016/s0959-4388(03)00103-x 12965288

[60] Sabesan R, Schmidt BP, Tuten WS, Roorda A. The elementary representation of spatial and color vision in the human retina. Sci Adv. 2016;2:e1600797–e1600797. doi: 10.1126/sciadv.1600797 27652339 PMC5023317

[61] Seifert M, Baden T, Osorio D. The retinal basis of vision in chicken. Semin Cell Dev Biol. 2020;106:106–115. doi: 10.1016/j.semcdb.2020.03.011 32295724

[62] Hellevik MH, Mardoum P, Hahn J, Yoshimatsu T. Ancient origin of the rod bipolar cell pathway in the vertebrate retina. bioRxiv. 2023. 10.1038/s41559-024-02404-w 38627529

[63] Kelber A, Vorobyev M, Osorio D. Animal colour vision—behavioural tests and physiological concepts. Biol Rev Camb Philos Soc. 2003;78:81–118. doi: 10.1017/s1464793102005985 12620062

[64] Potier S, Lieuvin M, Pfaff M, Kelber A. How fast can raptors see? J Exp Biol. 2020:223. doi: 10.1242/jeb.209031 31822552

[65] Seifert M, Roberts PA, Kafetzis G, Osorio DA, Baden T. Birds multiplex spectral and temporal visual information via retinal On–and Off–channels. Nat Commun. 2023; 14:5308. doi: 10.1038/s41467-023-41032-z 37652912 PMC10471707

[66] Mitkus M, Olsson P, Toomey MB, Corbo JC, Kelber A. Specialized photoreceptor composition in the raptor fovea. J Comp Neurol. 2017;529:2152–2163. doi: 10.1002/cne.24190 28199005 PMC6235456

[67] Kelber A, Yovanovich C, Olsson P. Thresholds and noise limitations of colour vision in dim light. Philos Trans R Soc B Biol Sci. 2017;372:20160065. doi: 10.1098/rstb.2016.0065 28193810 PMC5312015

[68] Passaglia CL, Troy JB. Impact of Noise on Retinal Coding of Visual Signals. J Neurophysiol. 2004;92:1023–1033. doi: 10.1152/jn.01089.2003 15071086 PMC5130336

[69] Thomas KN, Gower DJ, Bell RC, Fujita MK, Schott RK, Streicher JW. Eye size and investment in frogs and toads correlate with adult habitat, activity pattern and breeding ecology. Proc R Soc B Biol Sci. 2020;287:20201393. doi: 10.1098/rspb.2020.1393 32962540 PMC7542830

[70] Yovanovich CAM, Koskela SM, Nevala N, Kondrashev SL, Kelber A, Donner K. The dual rod system of amphibians supports colour discrimination at the absolute visual threshold. Philos Trans R Soc B Biol Sci. 2017. doi: 10.1098/rstb.2016.0066 28193811 PMC5312016

[71] Kojima K, Matsutani Y, Yamashita T, Yanagawa M, Imamoto Y, Yamano Y, et al. Adaptation of cone pigments found in green rods for scotopic vision through a single amino acid mutation. Proc Natl Acad Sci U S A. 2017;114:5437–5442. doi: 10.1073/pnas.1620010114 28484015 PMC5448186

[72] Lamb TD, Collin SP, Pugh EN Jr. Evolution of the vertebrate eye: Opsins, photoreceptors, retina and eye cup. Nat Rev Neurosci. 2007;8:960–976. doi: 10.1038/nrn2283 18026166 PMC3143066

[73] Hauzman E. Adaptations and evolutionary trajectories of the snake rod and cone photoreceptors. Semin Cell Dev Biol. 2020;106:86–93. doi: 10.1016/j.semcdb.2020.04.004 32359892

[74] Kojima K, Matsutani Y, Yanagawa M, Imamoto Y, Yamano Y, Wada A, et al. Evolutionary adaptation of visual pigments in geckos for their photic environment. Sci Adv. 2021;7:eabj1316. doi: 10.1126/sciadv.abj1316 34597144 PMC10938493

[75] Baden T, Schubert T, Chang LL, Wei T, Zaichuk M, Wissinger B, et al. A tale of two retinal domains: near-optimal sampling of achromatic contrasts in natural scenes through asymmetric photoreceptor distribution. Neuron. 2013;80:1206–1217. doi: 10.1016/j.neuron.2013.09.030 24314730

[76] Nadal-Nicolás FM, Kunze VP, Ball JM, Peng BT, Krisnan A, Zhou G, et al. True S-cones are concentrated in the ventral mouse retina and wired for color detection in the upper visual field. Elife. 2020;9:1–30. doi: 10.7554/eLife.56840 32463363 PMC7308094

[77] Gerkema MP, Davies WIL, Foster RG, Menaker M, Hut RA. The nocturnal bottleneck and the evolution of activity patterns in mammals. Proc Biol Sci. 2013;280:20130508. doi: 10.1098/rspb.2013.0508 23825205 PMC3712437

[78] Kumar S, Hedges SB. A molecular timescale for vertebrate evolution. Nature. 1998;392:917–920. doi: 10.1038/31927 9582070

[79] Arrese C, Dunlop SA, Harman AM, Braekevelt CR, Ross WM, Shand J, et al. Retinal structure and visual acuity in a polyprotodont marsupial, the fat-tailed dunnart (Sminthopsis crassicaudata). Brain Behav Evol. 1999;53:111–126. doi: 10.1159/000006588 10085478

[80] Hagen JFD, Roberts NS, Johnston RJ. The evolutionary history and spectral tuning of vertebrate visual opsins. Dev Biol. 2023;493:40–66. doi: 10.1016/j.ydbio.2022.10.014 36370769 PMC9729497

[81] Cowing JA, Arrese CA, Davies WL, Beazley LD, Hunt DM. Cone visual pigments in two marsupial species: the fat-tailed dunnart (Sminthopsis crassicaudata) and the honey possum (Tarsipes rostratus). Proc R Soc B Biol Sci. 2008;275:1491–1499. doi: 10.1098/rspb.2008.0248 18426754 PMC2602665

[82] Burger JR, George MA, Leadbetter C, Shaikh F. The allometry of brain size in mammals. J Mammal. 2019; 100(2):276–283. doi: 10.1093/jmammal/gyz043

[83] Smaers JB, Rothman RS, Hudson DR, Balanoff AM, Beatty B, Dechmann DKN, et al. The evolution of mammalian brain size. Sci Adv. 2021; 7(18):eabe2101. doi: 10.1126/sciadv.abe2101 33910907 PMC8081360

[84] Goetz J, Jessen ZF, Jacobi A, Mani A, Cooler S, Greer D, et al. Unified classification of mouse retinal ganglion cells using function, morphology, and gene expression. Cell Rep. 2022;40:111040. doi: 10.1016/j.celrep.2022.111040 35830791 PMC9364428

[85] Baden T, Berens P, Franke K, Roman-Roson M, Bethge M, Euler T. The functional diversity of mouse retinal ganglion cells. Nature. 2016:1–21. doi: 10.1038/nature16468 26735013 PMC4724341

[86] Westheimer G. The ON-OFF dichotomy in visual processing: From receptors to perception. Prog Retin Eye Res. 2007;26:636–648. doi: 10.1016/j.preteyeres.2007.07.003 17851105

[87] Boycott BB, Wassle H. Parallel processing in the mammalian retina—The Proctor Lecture. Invest Ophthalmol Vis Sci. 1999;40:1313–1327. 10359312

[88] Franke K, Berens P, Schubert T, Bethge M, Euler T, Baden T. Inhibition decorrelates visual feature representations in the inner retina. Nature. 2017;542:439–444. doi: 10.1038/nature21394 28178238 PMC5325673

[89] Pitkow X, Meister M. Decorrelation and efficient coding by retinal ganglion cells. Nat Neurosci. 2012;15:628–635. doi: 10.1038/nn.3064 22406548 PMC3725273

[90] Doi E, Gauthier JL, Field GD, Shlens J, Sher A, Greschner M, et al. Efficient Coding of Spatial Information in the Primate Retina. J Neurosci. 2012;32:16256–16264. doi: 10.1523/JNEUROSCI.4036-12.2012 23152609 PMC3537829

[91] Jun NY, Field GD, Pearson JM. Efficient coding, channel capacity and the emergence of retinal mosaics. bioRxiv. 2022:p. 2022.08.29.505726. doi: 10.1101/2022.08.29.505726 37168261 PMC10168625

[92] Atick JJ, Redlich AN. What Does the Retina Know about Natural Scenes? Neural Comput. 1992;4:196–210. doi: 10.1162/neco.1992.4.2.196

[93] Barlow HBH. Possible principles underlying the transformation of sensory messages. Sensory Communication. 1961:217–234. doi: 10.1080/15459620490885644

[94] Simoncelli EP, Olshausen BA. Natural image statistics and neural representation. Annu Rev Neurosci. 2001;24:1193–1216. doi: 10.1146/annurev.neuro.24.1.1193 11520932

[95] von Eugen K, Endepols H, Drzezga A, Neumaier B, Güntürkün O, Backes H, et al. Avian neurons consume three times less glucose than mammalian neurons. Curr Biol. 2022;32:4306–4313.e4. doi: 10.1016/j.cub.2022.07.070 36084646

[96] Hahn J, Monavarfeshani A, Qiao M, Kao A, Kölsch Y, Kumar A, et al. Evolution of neuronal cell classes and types in the vertebrate retina. bioRxiv. 2023:p. 2023.04.07.536039. doi: 10.1101/2023.04.07.536039 38092908 PMC10719112

[97] Field GD, Gauthier JL, Sher A, Greschner M, Machado TA, Jepson LH, et al. Functional connectivity in the retina at the resolution of photoreceptors. Nature. 2010;467:673–677. doi: 10.1038/nature09424 20930838 PMC2953734

[98] Buchsbaum G, Gottschalk A. Trichromacy, opponent colours coding and optimum colour information transmission in the retina. Proc R Soc Lond Ser B Contain Pap Biol Character R Soc G B. 1983;220:89–113. doi: 10.1098/rspb.1983.0090 6140684

[99] Roy S, Jun NY, Davis EL, Pearson J, Field GD. Inter-mosaic coordination of retinal receptive fields. Nature. 2021;10.1038/s41586-021–03317–5. doi: 10.1038/s41586-021-03317-5 33692544 PMC8049984

[100] Manookin MB, Patterson SS, Linehan CM. Neural Mechanisms Mediating Motion Sensitivity in Parasol Ganglion Cells of the Primate Retina. Neuron. 2018;97:1327–1340.e4. doi: 10.1016/j.neuron.2018.02.006 29503188 PMC5866240

[101] Kolb H, Marshak D. The midget pathways of the primate retina. Doc Ophthalmol. 2003:67–81. doi: 10.1023/a:1022469002511 12675488

[102] Peng Y-R, Shekhar K, Yan W, Herrmann D, Sappington A, Bryman GS, et al. Molecular Classification and Comparative Taxonomics of Foveal and Peripheral Cells in Primate Retina. Cell. 2019;176:1222–1237.e22. doi: 10.1016/j.cell.2019.01.004 30712875 PMC6424338

[103] Moskowitz-Cook A. The development of photopic spectral sensitivity in human infants. Vision Res. 1979;19:1133–1142. doi: 10.1016/0042-6989(79)90009-9 550571

[104] Skelton AE, Maule J, Franklin A. Infant color perception: Insight into perceptual development. Child Dev Perspect. 2022;16:90–95. doi: 10.1111/cdep.12447 35915666 PMC9314692

[105] Nagle MG, Osorio D. The tuning of human photopigments may minimize red-green chromatic signals in natural conditions. Proc Biol Sci. 1993;252:209–213. doi: 10.1098/rspb.1993.0067 8394581

[106] van Esch JA, Koldenhof EE, van Doorn AJ, Koenderink JJ. Spectral sensitivity and wavelength discrimination of the human peripheral visual field. J Opt Soc Am A. 1984;1:443–450. doi: 10.1364/josaa.1.000443 6726492

[107] Field GD, Sher A, Gauthier JL, Greschner M, Shlens J, Litke AM, et al. Spatial Properties and Functional Organization of Small Bistratified Ganglion Cells in Primate Retina. J Neurosci. 2007;27:13261–13272. doi: 10.1523/JNEUROSCI.3437-07.2007 18045920 PMC6673390

[108] Ghosh KK, Grünert U. Synaptic input to small bistratified (blue-ON) ganglion cells in the retina of a new world monkey, the marmoset Callithrix jacchus. J Comp Neurol. 1999;413:417–428. doi: 10.1002/(sici)1096-9861(19991025)413:3&lt;417::aid-cne5&gt;3.0.co;2-h 10502249

[109] Kim YJ, Peterson BB, Crook JD, Joo HR, Wu J, Puller C, et al. Origins of direction selectivity in the primate retina. Nat Commun. 2022;13:2862. doi: 10.1038/s41467-022-30405-5 35606344 PMC9126974

[110] Puller C, Manookin MB, Neitz J, Rieke F, Neitz M. Broad Thorny Ganglion Cells: A Candidate for Visual Pursuit Error Signaling in the Primate Retina. J Neurosci. 2015:35. doi: 10.1523/JNEUROSCI.4369-14.2015 25834063 PMC4381007

[111] Hepschke JL, Martin PR, Fraser CL. Short-Wave Sensitive (“Blue”) Cone Activation Is an Aggravating Factor for Visual Snow Symptoms. Front Neurol. 2021;12. Available from: doi: 10.3389/fneur.2021.697923 34489849 PMC8418220

[112] Slow intrinsic rhythm in the koniocellular visual pathway. Proc Natl Acad Sci U S A. [cited 2023 Jul 6]. Available from: https://www.pnas.org/doi/full/10.1073/pnas.1108004108. 21844334 10.1073/pnas.1108004108 PMC3167552

[113] Qiu Y, Zhao Z, Klindt D, Kautzky M, Szatko KP, Schaeffel F, et al. Natural environment statistics in the upper and lower visual field are reflected in mouse retinal specializations. Curr Biol. 2021. doi: 10.1016/j.cub.2021.05.017 34107304

[114] Szatko KP, Korympidou MM, Ran Y, Berens P, Dalkara D, Schubert T, et al. Neural circuits in the mouse retina support color vision in the upper visual field. Nat Commun. 2020;11:3481. doi: 10.1038/s41467-020-17113-8 32661226 PMC7359335

[115] Applebury ML, Antoch MP, Baxter LC, Chun LLY, Falk JD, Farhangfar F, et al. The Murine Cone Photoreceptor: A Single Cone Type Expresses Both S and M Opsins with Retinal Spatial Patterning. Neuron. 2000;27:513–523. doi: 10.1016/s0896-6273(00)00062-3 11055434

[116] Szél A, Röhlich P, Caffé AR, Juliusson B, Aguirre G, Van Veen T. Unique topographic separation of two spectral classes of cones in the mouse retina. J Comp Neurol. 1992;325:327–342. doi: 10.1002/cne.903250302 1447405

[117] Belušic G, Pirih P, Stavenga DG. A cute and highly contrast-sensitive superposition eye—the diurnal owlfly Libelloides macaronius. J Exp Biol. 2013;216:2081–2088. doi: 10.1242/jeb.084194 23431000

[118] Calderone J, Reese B, Jacobs G. Topography of photoreceptors and retinal ganglion cells in the spotted hyena (Crocuta crocuta). Brain Behav Evol. 2003 [cited 2014 Jun 20]. Available from: http://www.karger.com/Article/Abstract/73270. doi: 10.1159/000073270 14573992

[119] Tedore C, Nilsson DE. Avian UV vision enhances leaf surface contrasts in forest environments. Nat Commun. 2019;10:238. doi: 10.1038/s41467-018-08142-5 30670700 PMC6342963

[120] Nilsson D-E, Smolka J. Quantifying biologically essential aspects of environmental light. J R Soc Interface. 2021;18:rsif.2021.0184. doi: 10.1098/rsif.2021.0184 33906390 PMC8086911

[121] Cronin TW, Bok MJ. Photoreception and vision in the ultraviolet. J Exp Biol. 2016;219:2790–2801. doi: 10.1242/jeb.128769 27655820

[122] Baden T. Vertebrate vision: Lessons from non-model species. Semin Cell Dev Biol. 2020;106:1–4. doi: 10.1016/j.semcdb.2020.05.028 32532616

[123] Fraser B, DuVal MG, Wang H, Allison WT. Regeneration of Cone Photoreceptors when Cell Ablation Is Primarily Restricted to a Particular Cone Subtype. PLoS ONE. 2013:8. doi: 10.1371/journal.pone.0055410 23383182 PMC3559598

[124] Olsson P, Wilby D, Kelber A. Spatial summation improves bird color vision in low light intensities. Vision Res. 2017;130:1–8. doi: 10.1016/j.visres.2016.10.009 27845179

[125] Wilby D, Roberts NW. Optical influence of oil droplets on cone photoreceptor sensitivity. J Exp Biol. 2017. doi: 10.1242/jeb.152918 28314749 PMC5482973

