# Correction: From water to land: Evolution of photoreceptor circuits for vision in air

**DOI:** 10.1371/journal.pbio.3002588

**Published:** 2024-03-27

**Authors:** Tom Baden

Reference 1 is incomplete. The complete reference is: Baden T. Ancestral photoreceptor diversity as the basis of visual behaviour. Nat Ecol Evol. 2024;8:374–386. pmid:38253752.

The DOI for reference 1 is https://doi.org/10.1038/s41559-023-02291-7.

In the legend for Fig 3, a reference is omitted from the description of panel b. The correct sentence is: (b) Spectral sensitivity functions of chicken cones with oil droplets included (single cones based on ref [125], double cone principal member based on ref [126]).

The citation for reference 126 is: Wilby D. Optics and photoreception in the avian retina. PhD thesis, University of Bristol, 2015. Available from: https://doi.org10.13140/RG.2.2.18130.15043.
